# Temporal and Severity-Dependent Alterations in Plasma Extracellular Vesicle Profiles Following Spinal Cord Injury

**DOI:** 10.3390/cells14141065

**Published:** 2025-07-11

**Authors:** Jamie Cooper, Scott Tait Airey, Eric Patino, Theo Andriot, Mousumi Ghosh, Damien D. Pearse

**Affiliations:** 1 The Miami Project to Cure Paralysis, Department of Neurological Surgery, University of Miami Miller School of Medicine, Miami, FL 33136, USA; sxa2167@med.miami.edu (S.T.A.); emp245@med.miami.edu (E.P.); txa825@med.miami.edu (T.A.); mghosh@med.miami.edu (M.G.); dpearse@med.miami.edu (D.D.P.); 2 The Neuroscience Program, University of Miami Miller School of Medicine, Miami, FL 33136, USA; 3 The Interdisciplinary Stem Cell Institute, University of Miami Miller School of Medicine, Miami, FL 33136, USA

**Keywords:** spinal cord injury (SCI), small extracellular vesicles (sEVs), plasma biomarkers, injury severity, subacute phase, chronic phase, platelet activation

## Abstract

Spinal cord injury (SCI) triggers both local and systemic pathological responses that evolve over time and differ with injury severity. Small extracellular vesicles (sEVs), known mediators of intercellular communication, may serve as biomarkers reflecting these complex dynamics. In this study, we investigated whether SCI severity modulates the composition and abundance of circulating plasma-derived sEVs across subacute and chronic phases. Using a graded thoracic contusion model in mice, plasma was collected at defined timepoints post-injury. sEVs were isolated via size-exclusion chromatography and characterized using nanoparticle tracking analysis (NTA), transmission electron microscopy (TEM), and MACSPlex surface marker profiling. We observed an SCI-dependent increase in sEVs during the subacute (7 days) phase, most notably in moderate injuries (50 kdyne), with overall vesicle counts lower chronically (3 months). CD9 emerged as the predominant tetraspanin sEV marker, while CD63 and CD81 were generally present at low levels across all injury severities and timepoints. Surface sEV analysis revealed dynamic regulation of CD41^+^, CD44^+^, and CD61^+^ in the CD9^+^ sEV subset, suggesting persistent systemic signaling activity. These markers, traditionally associated with platelet function, may also reflect immune or reparative responses following SCI. Our findings highlight the evolving nature of sEV profiles after SCI and support their potential as non-invasive biomarkers for monitoring injury progression.

## 1. Introduction

Spinal cord injury (SCI) affects an estimated 250,000–500,000 individuals annually worldwide, according to the World Health Organization [1], resulting in long-term disability that profoundly impacts an individual’s quality of life. Beyond its personal toll, SCI presents itself as an ongoing global health crisis and imposes a significant socioeconomic burden with its high treatment costs and requirement for lifelong medical support [2].

The pathophysiology of SCI involves both primary and secondary injury mechanisms. Initial mechanical trauma induces localized physical damage at the injury site, followed by a subsequent cascade of secondary events leading to immune dysregulation, inflammation, oxidative stress, an exacerbation of neural cell death, and glial scarring [3,4,5,6]. Additionally, widespread systemic effects, including pulmonary, cardiovascular, and other organ-specific complications, are observed [7,8,9]. Such changes contribute to impaired tissue repair, increased susceptibility to infections, and prolonged inflammation, all of which negatively influence recovery trajectories [9,10,11,12]. Despite advances in subacute care and rehabilitation, protective or restorative therapeutics remain limited clinically due to the complex nature of SCI, highlighting the importance of gaining a deeper understanding of its multifaceted pathophysiology and developing more comprehensive treatment strategies.

Emerging evidence indicates that immune dysregulation after SCI extends beyond the central nervous system and involves complex systemic signaling mechanisms that remain incompletely understood [13]. Among the key mediators of intercellular communication are small extracellular vesicles (sEVs), lipid bilayer-enclosed particles secreted by all cell types. sEVs carry bioactive cargos, including proteins, RNAs, and lipids; the identities of which reflect the biological state of the parental cell at release. This cell-specific signaling plays a vital role in modulating immune responses and facilitating crosstalk between the central nervous system and peripheral organs in various disease contexts [14,15,16]. In addition to their potential use as therapeutic agents [17,18], including in SCI [17], recently sEVs have emerged as promising candidates for biomarker discovery due to their stability in biofluids and capacity to reflect the physiological state of the cells from which they are released [19]. Relative to SCI, alterations in plasma sEV composition and profiles may provide valuable insight into systemic immune dynamics and identify molecular signatures associated with injury severity. Severity-dependent biomarkers could enhance diagnostic precision and support the development of personalized therapeutic strategies aimed at improving recovery outcomes. Recent studies have demonstrated promise in using sEVs as biomarkers for SCI; however, the temporal dynamics and molecular characteristics of circulating sEVs in relation to specific injury severities remain poorly defined [20].

Studies have explored the potential of circulating biomarkers to predict the severity of SCI during the acute phase and to inform therapeutic decision-making. While much of this work has centered on free-circulating miRNAs or neural proteins, there is growing recognition that miRNAs and other cargos encapsulated within sEVs may provide a more robust and physiologically relevant means of stratifying injury severity [21]. Although a few studies have begun to investigate the inflammatory cargo of sEVs after SCI [22], variability in sEV isolation techniques and a lack of standardized methodologies have limited the comparability and interpretability of these findings. Moreover, comprehensive analyses that stratify plasma sEV profiles by injury severity, considering both quantitative and phenotypic characteristics, are still needed. Filling this critical gap is essential to establishing sEVs as reliable biomarkers for monitoring SCI severity and progression.

**Figure 1:**
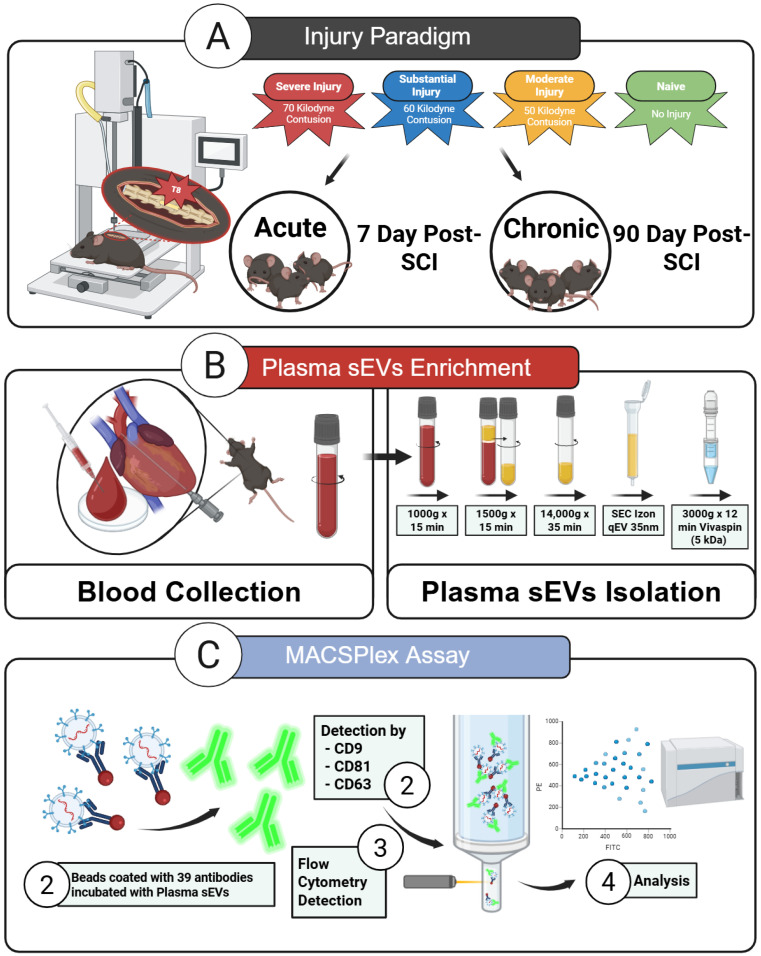
Experimental workflow for the isolation and analysis of plasma-derived sEVs after SCI. (**A**) Schematic representation of the SCI paradigm, showing graded contusion injuries of 50, 60, and 70 kilodynes (kdyn) at thoracic level T8, along with uninjured naïve controls. Plasma samples were collected at two key timepoints: subacute (7 days post-injury) and chronic (90 days post-injury). (**B**) Workflow for sEV enrichment from plasma. Perfusate blood was collected via cardiac puncture post-euthanasia and centrifuged to obtain plasma. sEVs were enriched using size-exclusion chromatography (SEC) with qEVoriginal 70 nm columns, enabling removal of soluble protein contaminants and isolation of vesicle-rich fractions. (**C**) Multiplex immunophenotyping of plasma-derived sEVs using the MACSPlex assay. Enriched vesicles were incubated with a bead-based panel containing 39 antibodies targeting immune and adhesion molecules. Surface marker detection was carried out using a fluorescent tetraspanin cocktail (CD63, CD81, CD9), and samples were analyzed by flow cytometry.

This study represents the first comprehensive investigation of circulating plasma-derived sEV profiles across multiple SCI severities and timepoints that span subacute and chronic phases. We performed multiparametric profiling of plasma-derived sEVs to identify early and late sEV surface signatures predictive of lesion magnitude (Figure 1). Using a graded T8 thoracic contusion SCI in mice, plasma was collected at either 7 days or 3 months after injury. Plasma sEVs were isolated via size-exclusion chromatography (SEC) and characterized for size and concentration by nanoparticle tracking analysis (NTA) and morphology by transmission electron microscopy (TEM). To evaluate sEV surface marker profiles, we employed MACSPlex immunophenotyping. By comparing sEV features across injury severities and phases, this study aimed to analyze sEV dynamics and identify potential biomarkers of systemic immune dysregulation and disease progression in SCI.

## 2. Materials and Methods

### 2.1. Animals

Adult female C57BL/6 mice were purchased from The Jackson Laboratory (Bar Harbor, ME, USA). Mice were selected by age: *Chronic* at ~88 days of age at SCI (range: 18.3–22.6 g; average: 21.1 g), ~180 days of age at blood collection; *Subaccute* at ~174 days of age at SCI (range: 20.3–27.6 g, average 23.9 g), ~181 days of age at blood collection. Blood collection from naïve mice was obtained at ~180 days of age. All animals were housed according to the *NIH* and *The Guide for the Care and Use of Animals* (ISBN: 0-309-15401-4, NRC 2011) standards [23], and all procedures involved in this study were approved by the Institutional Animal Care and Use Committee (Protocol # 22-099; approved 14 October 2022). Mice were housed in virus/antigen-free environments under diurnal lighting conditions, provided environmental enrichment, and allowed free access to food and water.

### 2.2. Experimental Group

A total of 43 adult female C57Bl/6J mice were utilized in both Subacute and Chronic Experimental groups. Mice were assigned to the following injury severity paradigms: (A) Severe SCI—70 kilodyne (n = 15); (B) Moderate–Severe SCI—60 kilodyne (n = 13); (C) Moderate SCI—50 kilodyne (n = 11); and (D) Naïve (n = 4).

### 2.3. Preoperative Procedure and Anesthetic Monitoring

Prior to SCI, animals were weighed and anesthetized using a mixture of 2% isoflurane and 30% oxygen. The depth of anesthesia was monitored by assessing the corneal reflex and evaluating the withdrawal response to hind limb or paw/whisker stimulation, both initially and at 15 min intervals until recovery. The dorsal aspect of the back above the desired injury location (Thoracic vertebra T8) was shaved and prepped with chlorhexidine scrub and solution. During surgery, the animal was placed on a sterile drape with a feedback-controlled heating blanket underneath to maintain a core body temperature of 37.5 degrees Celsius (as monitored by a rectal thermometer). OphtHAvet (Dechra Veterinary Products, Overland Park, KS, USA) was applied to the eyes to prevent drying.

### 2.4. Thoracic Contusion Injury Paradigm

A laminectomy at the T8 vertebral level was performed on anesthetized mice. Muscle and connective tissue were dissected from the transverse processes of the T7 and T9 vertebrae to facilitate secure fixation between the T7/T9 vertebral segments and the clamping platform (Infinite Horizons (IH) SCI device, Precision Systems & Instrumentation, Lexington, KY, USA). A laminectomy was performed at the level of the T8 vertebra, involving removal of the spinous process and underlying laminar bone to create a ~1.5 mm diameter fenestration, providing direct access to the dorsal surface of the spinal cord and permitting unobstructed contact with the impactor tip (~1.25 mm diameter). Care was taken during the laminectomy to maintain uniformity in size and lateral extent of exposure. Spinal injury models targeted the following impact values: (A) Severe, 70 kdyne; (B) Moderate–Severe, 60 kdyne; and (C) Moderate, 50 kdyne. Injury parameters were recorded automatically during injury [24]. Following injury, the back muscles were closed via absorbable sutures, and the skin was closed with mouse wound clips.

### 2.5. Post-Operative Procedure

Following surgery, mice recovered from anesthesia in a warmed cage and were administered the following: Buprenorphine XR (3.25 mg/kg, subcutaneous) administered once directly following surgery; Gentamicin (5 mg/kg, subcutaneous) administered daily for 7 days post-surgery; and Lactated Ringer’s Solution (up to 1 mL, subcutaneous) BID as needed for sufficient hydration for 7 days. Bladders were manually expressed by gentle abdominopelvic compression twice daily (Crede method) until bladder function returned. Animals were provided with free access to food, water, and gel packs or water bottles equipped with long curved sipper tubes for those lacking hindlimb functionality.

### 2.6. Pre Hoc Criteria and Exclusions

Animals were excluded from this study based on predefined criteria. Exclusions included the following: injury force exceeding ± 2 standard deviations from the group average (n = 4); impact graphs indicating contact of the impactor with bone, defined according to the IH User Manual by an abnormal time–force curve (n = 3); and mortality during the post-survival period (n = 4). Therefore, the total number of animals excluded across cohorts was n = 10. Further exclusions were made based on NTA; samples with particle concentrations below 1 × 10^9^ particles/mL were excluded (n = 7). This threshold reflected the minimum concentration required to ensure reliable and interpretable downstream MACSPlex analysis.

### 2.7. Basso Mouse Scale (BMS) Locomotor Assessment

Locomotor function following spinal cord injury (SCI) was evaluated using the Basso Mouse Scale (BMS), a validated 9-point scoring system ranging from 0 (complete hindlimb paralysis) to 9 (normal locomotion). BMS scoring was performed by two blinded observers to ensure objective assessment. Mice were placed individually in an open field and allowed to move freely for 4 min while their hindlimb movements were observed and graded based on joint movement, paw placement, coordination, and trunk stability. For the subacute cohort, BMS scoring was conducted at 24 h and 7 days post-injury at the survival endpoint, prior to euthanasia and blood collection. Groups included naïve (uninjured), sham-operated, and SCI animals subjected to contusion injuries at 50, 60, or 70 kdyne. For the chronic cohort, BMS scores were recorded at 24 h and then weekly for 10 weeks post-injury to monitor long-term locomotor function.

### 2.8. Blood Collection

At the experimental endpoint, animals were euthanized via CO2 inhalation followed by thoracotomy to expose the thoracic cavity and heart. A blunt-tipped needle was inserted into the left ventricle and advanced toward the ascending aorta to enable transcardial perfusion. Sterile 0.9% saline (4 mL total; 8 mL/min) was perfused to clear the vasculature and displace systemic blood [25]. The animal was positioned to facilitate drainage directly into a 0.5 M EDTA-coated Petri dish. Perfusate blood was collected using S-Monovette^®^ 1.2 mL K3E blood collection syringes (22G; Sarstedt, Germany) and immediately placed on ice to preserve sample integrity. The timing of blood collection was performed so that all animals were of the same age (180 ± 2 days).

### 2.9. Platelet Free Plasma Preparation and Small Extracellular Vesicle Isolation

Perfusate blood was collected into EDTA-coated tubes and immediately processed. Samples were centrifuged at 1000× *g* for 15 min at 4 °C to separate plasma from cellular components. The upper plasma layer was carefully transferred to a clean microcentrifuge tube and centrifuged again at 1500× *g* for 15 min at 4 °C to remove residual cells and debris and stored at −80 °C as platelet-free plasma. Size-exclusion chromatography (SEC) was performed using qEVoriginal/70 nm columns (Izon Science Ltd., Christchurch, New Zealand) according to the manufacturer’s instructions. Columns were first equilibrated with sterile PBS. Subsequently, thawed platelet-free plasma was centrifuged again at 14,000× *g* for 35 min at 4 °C. The supernatant was carefully loaded onto each column, and fractions were collected into pre-chilled 1.5 mL microcentrifuge tubes and maintained on ice. A total of 15 fractions were collected per sample. NTA was used to determine particle size distribution and concentration across all collected fractions. In parallel, total protein content was assessed using the Pierce™ BCA Protein Assay Kit (Thermo Scientific, Rockford, IL, USA) following the manufacturer’s protocol. Based on NTA and protein quantification, sEV-rich fractions typically eluting between fractions 7 and 10, as recommended by the manufacturer, were identified and pooled. The pooled sEV-containing fractions were subsequently concentrated using a Vivaspin^®^ 2 centrifugal filter unit (5 kDa molecular weight cut-off; Sartorius, Göttingen, Germany) by centrifugation at 3000× *g* for 15 min or until the final volume was reduced to approximately 200 µL. Concentrated sEV samples were processed immediately for downstream applications.

### 2.10. Nanoparticle Tracking Analysis (NTA)

Size distribution and particle concentration of isolated sEVs were determined using NTA on the NanoSight NS300 platform (Malvern Panalytical Ltd., Malvern, UK). Samples were diluted in sterile, 0.22 μm filtered PBS to reach optimal measurement concentration (100–300 particles/frame). All measurements were conducted at room temperature, with each sample analyzed in five technical replicates. For each replicate, 30 frames were captured over a 30 s acquisition period using a detection threshold of 5. Data were processed using NanoSight NTA software (version 3.4 Build 3.4.4; Malvern Panalytical Ltd, Malvern, UK), and results were reported as mean particle concentration (particles/mL) and modal particle diameter (nm) per sample.

### 2.11. Transmission Electron Microscopy (TEM)

The sEV samples were prepared for ultrastructural imaging using transmission electron microscopy. A small volume of each sEV preparation was adsorbed onto formvar-coated carbon copper grids (200 mesh) and incubated for 30 min. Grids were then gently rinsed with phosphate buffer, followed by double-distilled water to remove unbound material. Fixation was performed using 2% glutaraldehyde, and samples were subsequently contrasted with 2% aqueous uranyl acetate. Prepared grids were protected from light and allowed to air-dry overnight. Imaging was performed using a JEM-1400 transmission electron microscope (JEOL Ltd., Peabody, MA, USA) operated at 80 kV. Images were captured using an AMT BioSprint 12 CCD camera system (Advanced Microscopy Techniques, Danvers, MA, USA).

### 2.12. Multiplex Bead-Based Flow Cytometric Analysis of sEV Surface Proteins by MACSPlex Exosome Kit Mouse

MACSPlex Exosome Kit, Mouse (Miltenyi Biotec, Bergisch Gladbach, Germany), is a multiplex bead-based flow cytometry assay that enables the semi-quantitative detection of 37 distinct surface markers on sEVs simultaneously (Supplementary Table S1). SEC isolated plasma-derived sEVs were used at a concentration of 1 × 10^9^ particles/mL, as determined by NTA. Samples with particle concentrations below this threshold were excluded from analysis, as this concentration represents the minimum required for reliable and reproducible bead-based detection in the MACSPlex assay. The sEV suspension was incubated with MACSPlex exosome capture beads under gentle agitation (450 rpm) at room temperature (RT) in a light-protected environment. To confirm successful binding of sEVs to the beads, fluorochrome-conjugated detection antibodies against the tetraspanins CD9, CD63, and CD81 were used. Following incubation, samples were washed with MACSPlex buffer and centrifuged at 3000× *g* for 5 min at RT. Buffer-only controls were included for each sample to determine background binding levels. After a final wash, events were assessed using a CytoFLEX flow cytometer (Beckman Coulter, Brea, CA, USA). Data were analyzed using FlowJo v10.10.0. Bead populations were gated based on their fluorescence profiles in the PE and FITC channels (Supplementary Figure S1). Background fluorescence from buffer-only was subtracted, and resulting values were normalized to the mean events of CD9 to determine the relative expression of each marker. The tetraspanin and sEV marker CD9 was used for normalization because of its consistently high expression compared to the very low abundance of the other tetraspanins, CD63 and CD81, across samples.

### 2.13. Statistical Analysis

GraphPad Prism v10 software was used for all statistical analyses, with data presented as mean ± SEM. Normality was assessed using the Shapiro–Wilk test. One-way ANOVA followed by Tukey’s multiple comparisons test was used to compare more than two groups with a single independent variable. For comparisons involving two independent variables (e.g., timepoint and injury severity), two-way ANOVA followed by Tukey’s post hoc test was applied. For comparisons between two groups, an unpaired *t*-test with Welch’s correction was used for parametric data, while the Mann–Whitney U test was applied for non-parametric data. Each experiment was performed in biological triplicate, and all statistical tests are specified in the figure legends. Significance levels are indicated as * *p*  <  0.05, ** *p*  <  0.01, *** *p*  <  0.001, and **** *p*  <  0.0001.

## 3. Results

### 3.1. Validation of Injury Severity Using Impactor Force Profiles and Functional BMS Scoring

**Figure 2:**
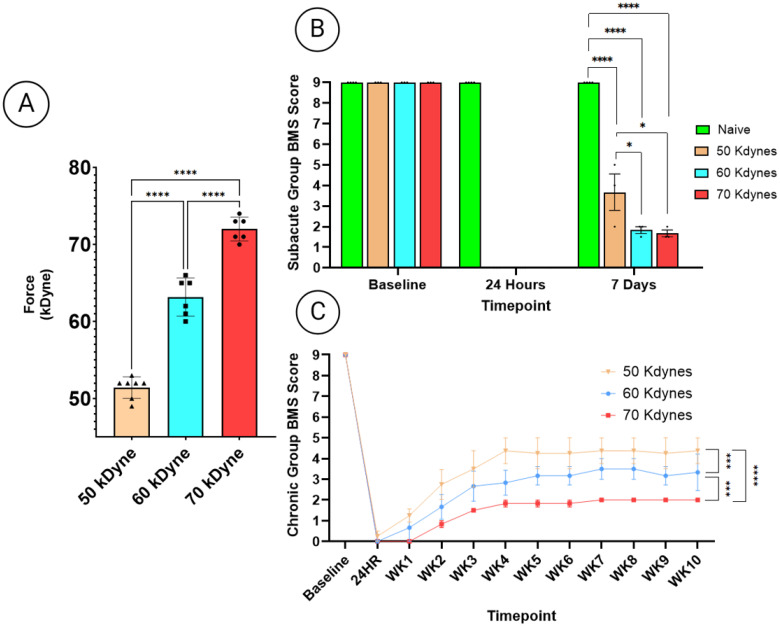
(**A**) Quantification of impact force at time of SCI for both subacute and chronic cohorts, demonstrating the distinct force profiles for the 50, 60, and 70 kdyne injury groups. (**B**) BMS locomotor scores for the subacute cohort, comparing naïve and SCI animals (50, 60, and 70 kdyne) at 24 h and 7 days post-injury. (**C**) BMS locomotor scores over 10 weeks for chronic SCI groups (50, 60, and 70 kdyne). Two-way ANOVA followed by Tukey’s multiple comparisons test was used to assess statistical significance across groups at the corresponding timepoints of blood collection. Significance is indicated as follows: * *p* < 0.05, *** *p* < 0.001, **** *p* < 0.0001. All experiments were performed in triplicate; error bars represent the standard error of the mean.

To confirm the consistency of the SCI and the severity of injury, two independent measures were employed: mechanical impact force values and BMS locomotor scoring. First, force impact values recorded during injury induction showed clear, statistically significant separation between the 50, 60, and 70 kdyne groups, validating the mechanical distinction between the graded injury severities (*p* < 0.0001) (Figure 2A). To complement the biomechanical data, functional assessments were performed using the BMS scale. For animals assigned to the subacute cohort (Figure 2B), BMS scoring was conducted at 24 h and again at 7 days post-injury, prior to euthanasia and blood collection. Naïve animals consistently demonstrated the highest BMS score compared to all injured groups (50, 60, and 70 kdyne) (*p* < 0.0001), confirming functional impairment as a result of SCI. BMS scores for the 50 kdyne group were also significantly higher than those for the 60 kdyne and 70 kdyne groups (*p* < 0.05), while no significant difference was observed at 7 days after SCI between the 60 kdyne and 70 kdyne groups. In the chronic cohort, BMS scoring was performed weekly over a 10-week period (Figure 2C). BMS scores for the 50 kdyne group were significantly higher than both the 60 kdyne (*p* < 0.001) and 70 kdyne groups (*p* < 0.0001), while the 60 kdyne BMS scores were also significantly higher than the 70 kdyne group (*p* < 0.001).

### 3.2. Characterization of Plasma-Derived sEVs Across Spinal Cord Injury Severities and Stages

**Figure 3:**
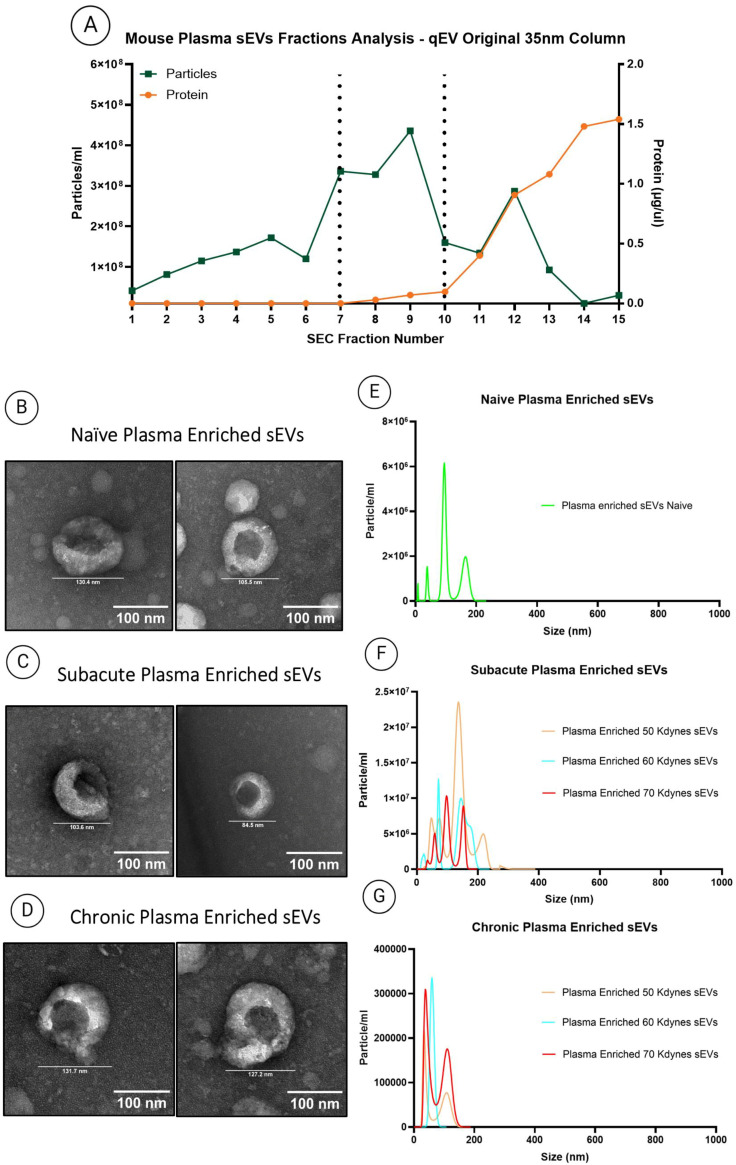
(**A**) Identification of sEV-rich plasma fractions using NTA and protein quantification following SEC. Fractions exhibiting low protein content and high particle concentration were pooled together and selected for downstream analysis. (**B**–**D**) TEM images showing representative sEV morphology from plasma of naïve (**B**), 70 kdyne and 50 kdyne subacute SCI (**C**), and chronic SCI (**D**) mice. (**E**–**G**) NTA profiles of plasma-derived sEVs from naïve (**E**), subacute SCI (**F**), and chronic SCI (**G**) groups. All experiments were performed in triplicate, with error bars representing the standard error of the mean. One-way ANOVA followed by Tukey’s multiple comparison test was used to assess statistical significance.

To investigate circulating sEVs in the context of SCI, plasma was collected from naïve mice and from animals subjected to graded contusion injuries at subacute (7 days post-injury) and chronic (90 days post-injury) time points. SEC isolated plasma sEVs were characterized in accordance with MISEV guidelines [26] using NTA and protein quantification to identify sEV-enriched fractions (Figure 3A). Fractions with high particle counts and low protein content were selected to minimize plasma protein contamination. TEM analysis revealed cup-shaped, negatively stained vesicles within the 30–150 nm size range in naïve, subacute, and chronic samples across all injury severities (Figure 3B–D). These findings were supported by NTA, which showed consistent sEV-like size distributions across groups (Figure 3E–G).

### 3.3. CD9 Is the Most Abundant Tetraspanin on Plasma-Derived sEVs Across Injury Severities and Stages

**Figure 4:**
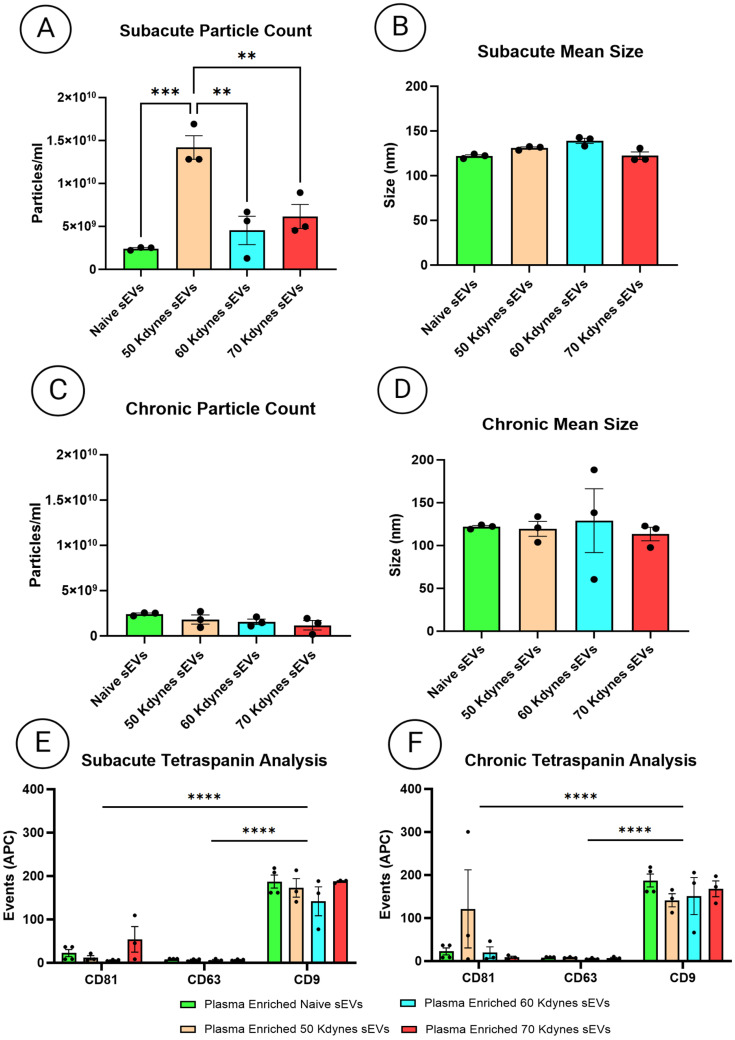
(**A**,**B**) NTA quantification of particle concentration in plasma-derived sEVs from naïve and injured mice in the subacute (**A**) and chronic (**B**) phases post-SCI. (**C**,**D**) NTA comparison of mean particle size for naïve versus injured plasma-derived sEVs in the subacute (**C**) and chronic (**D**) phases. One-way ANOVA followed by Tukey’s multiple comparison test was used to assess statistical significance. (**E**,**F**) MACSPlex analysis of tetraspanins (CD81, CD63, CD9) on plasma-derived sEVs from naïve and subacutely injured mice (**E**) and from naïve and chronically injured mice (**F**). Two-way ANOVA followed by Tukey’s multiple comparisons test was used to assess significance across tetraspanin markers. All experiments were performed in triplicate, with error bars representing the standard error of the mean. Significance is indicated as follows: ** *p*  < 0.01, *** *p*  < 0.001, **** *p*  < 0.0001. All experiments were performed in triplicate; error bars represent the standard error of the mean.

To assess particle concentration, size distribution, and surface tetraspanin composition of plasma-derived sEVs following SCI, we performed NTA and bead-based MACSPlex profiling. NTA revealed a 6-fold increase in particle concentration in sEV-enriched plasma fractions from subacutely injured 50 kdyne mice compared to naïve controls (*p* < 0.01). Moreover, particle concentration in the subacute 50 kdyn group was approximately 5-fold higher than in the 60 kdyne group and 3-fold higher than in the 70 kdyne group (*p* < 0.01) (Figure 4A). No significant changes in particle concentration or size distribution were observed in the other subacute injury groups or in any of the chronic SCI samples (Figure 4B–D). Next, we analyzed surface expression of classical sEV tetraspanin markers CD81, CD63, and CD9 using MACSPlex. Across both subacute and chronic phases, CD9 consistently emerged as the most abundant sEV marker in plasma-derived sEVs, regardless of injury severity (*p* < 0.0001). CD81 was detected at lower levels, whilst CD63 expression was undetectable in all groups (Figure 4E,F).

### 3.4. MACSPlex Profiling of Plasma-Derived sEVs Across Injury Severities in the Subacute Phase

**Figure 5:**
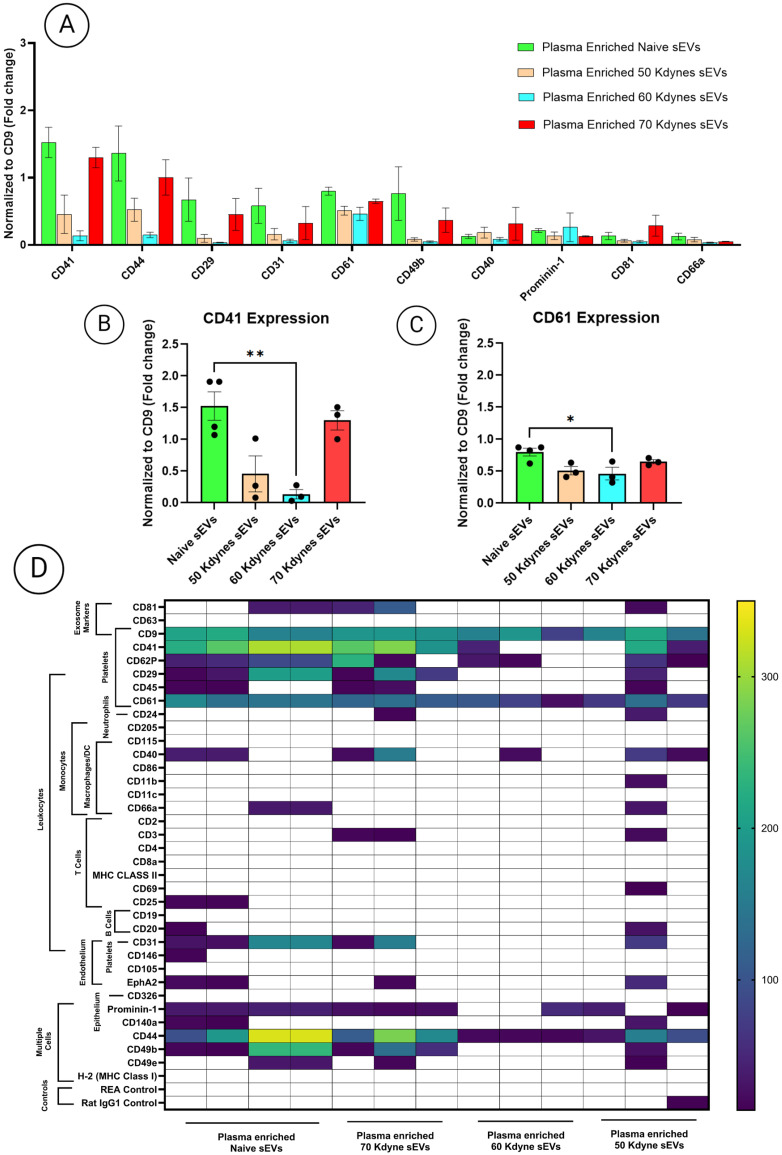
(**A**) MACSPlex analysis of plasma-derived sEVs at the subacute phase post-SCI, showing the top 10 most abundantly detected surface markers (normalized to CD9) across naïve and injury severity groups (50, 60, and 70 kdyne). (**B**,**C**) Quantitative comparisons of CD41 (**B**) and CD61 (**C**) binding across groups. (**D**) Summary heatmap of MACSPlex results illustrating relative expression patterns across the four groups. One-way ANOVA followed by Tukey’s multiple comparisons test was used to assess statistical significance. All experiments were performed in triplicate; data are presented as mean ± SEM. Significance levels: * *p*  <  0.05, ** *p*  <  0.01.

Given that CD9 was consistently the most abundantly detected tetraspanin across all experimental conditions, surface marker intensities obtained from MACSPlex analysis were normalized to CD9 expression (Supplementary Figure S2). This allowed for an accurate comparison of sEV surface profiles between groups by controlling for variations in particle number and tetraspanin content (Figure 5A–D). Initial surface marker profiling revealed consistent, high binding intensities for platelet-associated markers, specifically CD41 and CD61, in plasma-derived sEVs from the naïve group. Quantitative analysis demonstrated that CD41 expression was markedly reduced in injured animals, with an 11.2-fold decrease observed in the 60 kdyne group compared to naïve controls (*p* < 0.01). Moreover, a statistically significant reduction in CD61 expression was also identified, with levels 1.7-fold lower in the 60 kdyne group compared to naïve (*p* < 0.05). No statistically significant differences in CD41 or CD61 expression were detected between the 60 and 70 kdyne groups, or among any of the injured conditions.

### 3.5. MACSPlex Profiling of Plasma-Derived sEVs Across Injury Severities in the Chronic Phase

**Figure 6:**
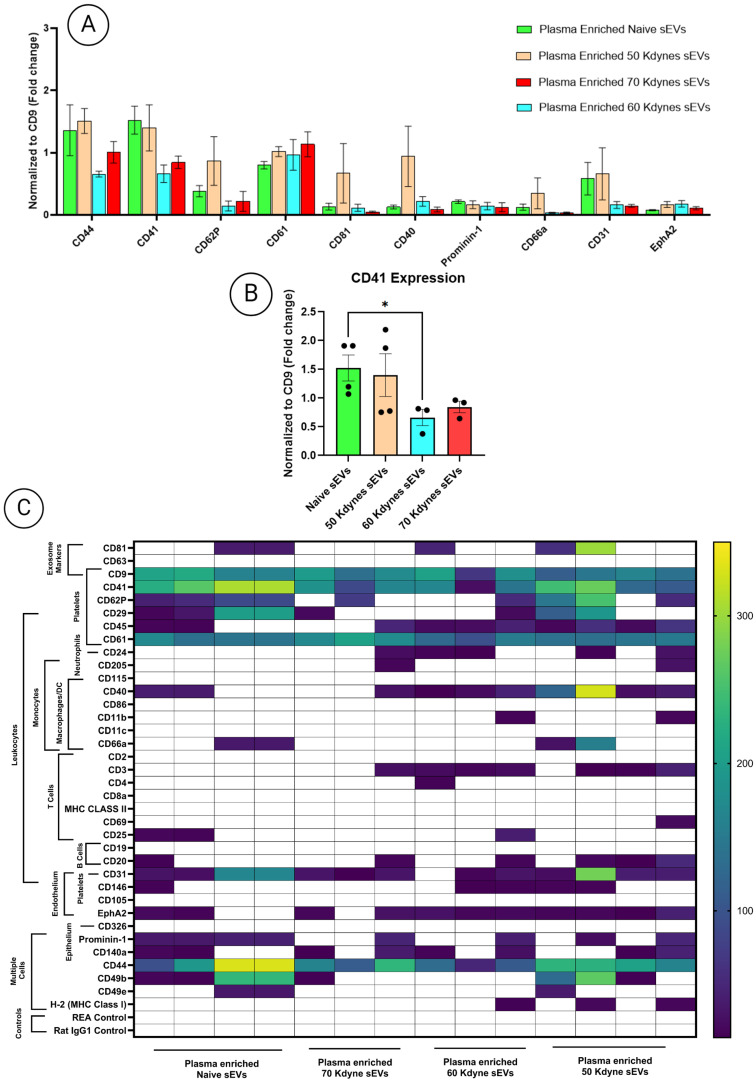
(**A**) MACSPlex analysis of plasma-derived sEVs at the chronic phase post-SCI, showing the top 10 most abundantly detected surface markers (normalized to CD9) across naïve and injury severity groups (50, 60, and 70 kdyne). (**B**) Quantitative comparisons of CD41 binding across groups. (**C**) Summary heatmap of MACSPlex results illustrating relative expression patterns across the four groups. One-way ANOVA followed by Tukey’s multiple comparisons test was used to assess statistical significance. All experiments were performed in triplicate; data are presented as mean ± SEM. Significance levels: * *p*  <  0.05.

To investigate whether surface marker alterations observed in the subacute phase persisted long-term, MACSPlex analysis was repeated at 90 days post-injury. Among all markers evaluated, CD41 continued to show reduced expression in injured groups relative to naïve controls, with a statistically significant 2.3-fold decrease in the 60 kdyne group (*p* < 0.05; Figure 6A–C). No other significant differences in CD41 expression were observed among the remaining injury groups, and no additional MACSPlex markers showed differential expression across conditions.

### 3.6. Comparative Profiling of Plasma-Derived sEVs Between Subacute and Chronic Phases Across Injury Severities

**Figure 7:**
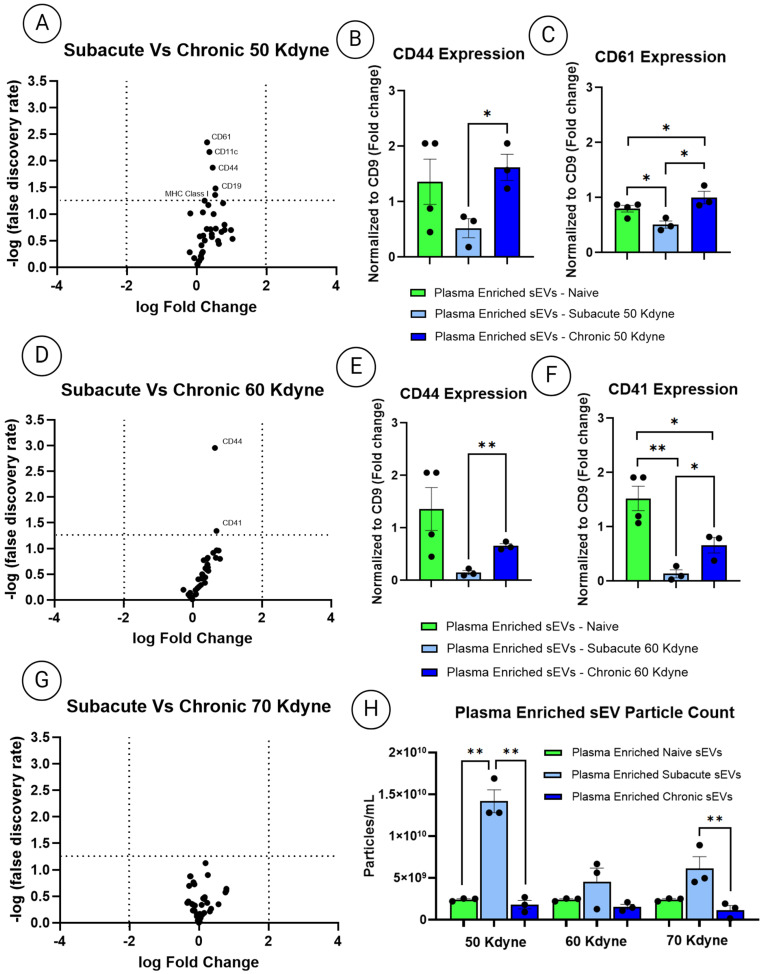
(**A**) Comparative analysis of plasma-enriched sEVs derived from subacute and chronic SCI across different injury severities. (**A**–**C**) Volcano plots illustrating differentially expressed surface markers in MACSPlex analysis between acute and chronic sEVs from mice with 50 kdyne injuries, including CD44 and CD61. (**D**–**F**) Mice with 60 kdyne injuries, including differences in expression of CD44 and CD41. (**G**) Mice with 70 kdyne, no differences observed. (**H**) Particle concentration comparisons of plasma-derived sEVs between acute and chronic timepoints across injury severities, as measured by NTA. One-way ANOVA followed by Tukey’s multiple comparisons test was used to assess group significance. All experiments were performed in triplicate; data are presented as mean ± SEM. Significance levels: * *p*  <  0.05, ** *p*  <  0.01.

To compare surface marker expression profiles of plasma-derived sEVs across injury severities and timepoints, volcano plots were generated using CD9-normalized MACSPlex data, analyzed via unpaired two-tailed *t*-tests. Results are presented as log_2_ fold-change versus –log_10_(*p*-value), with differential expression defined by a threshold of *p*  <  0.05 and an average log_2_ fold-change > 2. Initial comparisons between subacute and chronic sEVs in the 50 kdyne group revealed five significantly altered markers: CD61, CD11c, CD44, CD19, and MHC Class I. However, CD11c, CD19, and MHC Class I had low mean expression intensities (<0.2) and were excluded from interpretation to avoid overrepresenting background noise (Supplementary Figure S3). CD44 and CD61 remained prominent, with 3-fold and 2-fold increases, respectively, in plasma sEVs from chronic SCI compared to subacute (*p*  <  0.05) (Figure 7A–C). When comparing naïve and injured animals, CD44 expression remained unchanged, whereas CD61 showed dynamic changes across timepoints. Specifically, CD61 was 2.6-fold higher in naïve animals compared to the subacute group, but increased modestly in the chronic phase, showing a 1.2-fold elevation relative to naïve controls (*p*  <  0.05). In the 60 kdyne group, chronic-phase sEVs exhibited a significant 4.4-fold increase in CD44 expression (*p*  <  0.01) and a 4.9-fold increase in CD41 (*p*  <  0.05) compared to subacute sEVs (Figure 7D–F). Relative to naïve sEVs, chronic samples from this group also showed markedly increased CD44 and CD41 binding, emphasizing a dynamic surface profile shift across timepoints. While CD44 levels differed between naïve and injured phases, CD41 showed a pronounced decline post-injury, demonstrating an 11.2-fold higher expression in naïve sEVs compared to subacute (*p*  <  0.01), and a 2.3-fold higher expression compared to chronic sEVs (*p*  <  0.05). Comparisons were also made between the 70 kdyne group; however, no significant differences were observed (Figure 7G). Additionally, NTA analysis revealed distinct trends in sEV concentrations across severity groups and timepoints (Figure 7H). Subacute-phase sEVs from the 50 kdyne group exhibited a 5.8-fold increase in particle count relative to naïve, whilst a 7.8-fold difference from chronic-phase sEVs (*p*  <  0.01). The 70 kdyne group showed a 5.3-fold increase in subacute-phase sEVs in comparison to chronic-phase sEVs (*p*  <  0.01). Whilst no significant change in particle concentration was observed in the 60 kdyne group at the subacute phase.

## 4. Discussion

This study demonstrates that SCI induces dynamic changes in both the quantity and surface profiles of plasma-enriched sEVs, which vary according to injury severity and phase (subacute vs. chronic). To our knowledge, this is the first systematic profiling of plasma sEVs in a murine SCI model incorporating both temporal and severity-dependent analyses. By characterizing sEV surface marker expression across time points, we identify CD41^+^, CD44^+^, and CD61^+^ subpopulations as potential indicators of SCI progression, offering insight into both systemic inflammatory status and potential regenerative activity.

In the subacute phase, we observed a significant increase in circulating sEVs in 50 kdyn injured animals compared to both naïve controls and the more severely injured 60 and 70 kdyn groups, as confirmed via NTA. This elevated sEV abundance aligns with a less severe injury profile, as supported by significantly higher BMS scores in the 50 kdyn group relative to the others, indicating greater tissue preservation, more intact vasculature network at the injury site and retained locomotor function, which likely supports sustained sEV production and systemic dissemination in the blood. In contrast, more severe injuries likely involve greater vascular and cellular disruption, which could hinder sEV release into the circulation despite ongoing biogenesis [27,28]. Although sEV levels appeared lower in the 60 and 70 kdyn groups, this is unlikely to reflect reduced production, as DNA damage and stress are known to stimulate sEV release [29,30]. Instead, impaired vascular transport, local sequestration at the lesion, or enhanced clearance may contribute. Supporting this, previous SCI models have shown decreased plasma sEVs despite increased expression of EV biogenesis markers at the lesion site [22]. Interestingly, studies in traumatic brain injury (TBI) have reported elevated levels of CNS injury-related proteins (e.g., GFAP, NfL, UCH-L1) within sEVs in patients with more severe injuries [31]. However, these studies focus on sEV cargo, not total sEV abundance, and involve diffuse or focal brain injury in polytrauma patients where blood–brain barrier dynamics differ significantly from SCI. Therefore, our findings are not contradictory but may instead highlight tissue-specific responses, the role of vascular preservation in sEV dissemination, and the importance of distinguishing between sEV quantity and cargo. During the chronic phase, sEV counts declined across all groups and were no longer significantly different from naïve levels, potentially indicating a resolution of the acute response and return toward homeostasis.

Beyond changes in abundance, the composition and cellular origin of circulating sEVs also evolve following SCI, reflecting dynamic interactions between immune and neural systems. For example, following SCI, disruption of the blood–brain/spinal cord barrier (BBB/BSCB) permits infiltration of immune cells and peripheral activation of astrocytes and microglia/macrophages [22,32,33,34]. These cell types release sEVs carrying cargo reflective of the injury milieu. Notably, the heterogeneity of circulating sEVs complicates precise source attribution, especially in plasma, where both CNS and peripheral cells contribute to the sEV pool [22]. However, shifts in surface marker expression may offer indirect clues. Our use of the MACSPlex platform to assess sEV surface markers aligns with prior studies that have profiled sEVs under various pathological conditions [35,36]. For example, we observed variable CD81 expression across injury conditions, while not statistically significant, this trend aligns with reports of CD81 upregulation in microglia and downregulation in astrocytes, supporting a cell-type-influenced sEV signature [37]. CD63 was notably absent across our samples, whilst CD9 was abundant. This finding is consistent with previous reports describing undetectable CD63, whilst increased levels of CD9 in mouse plasma-derived sEVs, in contrast to cell line-derived sEVs [35]. Interestingly, comparisons of human plasma-enriched sEVs to those from mice show elevated CD63 and CD9 expression, but no detectable CD81, reinforcing the idea that tetraspanin abundance may be influenced by both sample origin and species. Similarly, robust detection of platelet-associated markers such as CD41^+^, CD44^+^, and CD61^+^ in our dataset mirrors previous mouse plasma findings using the MACSPlex assay [35].

The chronic persistence of specific sEV subtypes, particularly CD41^+^, CD44^+^, and CD61^+^ vesicles, after SCI compared to naïve, also suggests continued biological activity post-injury. These markers are commonly associated with platelet-derived sEVs but may also reflect activation of CNS-resident cells or peripherally mobilized inflammatory cells [38,39]. CD41 and CD61, components of the integrin complex, are traditionally linked to platelet function and coagulation, yet they are increasingly implicated in sEV-mediated cytokine modulation [40,41]. Their downregulation in the subacute phase and subsequent upregulation during the chronic phase, especially in moderate injuries, may reflect a biphasic immune response. Initial vesicle depletion could represent vesicle consumption or sequestration during the early inflammatory surge, while later recovery may coincide with ongoing low-grade inflammation. Indeed, persistent microglial activation and chronic neuroinflammation have been described months after SCI [42]. CD44, a cell adhesion molecule involved in inflammation and tissue remodeling [43], was also elevated during the chronic phase. Beyond its role in leukocyte trafficking, CD44 is expressed on neural stem-like cells [44], including ependymal cells lining the central canal, which proliferate after SCI and contribute to repair [45,46,47]. The increased abundance of CD44^+^ sEVs may therefore reflect both chronic inflammation and tissue remodeling efforts.

Several studies have aimed to establish more reliable biomarkers for SCI to support diagnosis, monitoring, and therapeutic evaluation. In our study, distinct sEV surface marker profiles such as CD41^+^, CD44^+^, and CD61^+^ were identified, showing injury severity- and phase-dependent dynamics. These findings highlight their potential utility as biomarkers reflective of injury progression. Similarly, a separate study using a human-specific MACSPlex panel, which includes a distinct set of antibodies from the mouse kit used in our study, identified four sEV markers, CD47, CD56, CD68, and ADAM17, that distinguished traumatic SCI (tSCI) patients from controls [20]. Notably, CD47^+^ sEVs correlated with neurological impairment, underscoring their value as potential biomarkers for tSCI. Several factors may contribute to the differing marker profiles between the studies for those antibodies that were present in both human and mouse kits, species differences in protein expression (e.g., CD56/NCAM is not reliably expressed in mice), variations in sEV isolation methodology (our study used Izon qEV columns versus Exo-Spin™ columns), and differing study aims, as our model was designed to systematically assess how subtle changes in SCI severity alter systemic sEVs over time. While both studies support the potential of sEVs as biomarkers, our findings emphasize the importance of incorporating injury severity and temporal dynamics into future biomarker discovery pipelines to enhance their clinical relevance.

Our findings reinforce the concept of a temporally dynamic, injury-responsive sEV landscape following SCI. sEVs are not passive byproducts but active participants in the evolving injury response, with the potential to either exacerbate or ameliorate damage depending on their cargo and cellular origin. The persistent presence of specific sEV subpopulations during the chronic phase supports the hypothesis of sustained vesicle-mediated signaling well beyond the subacute injury window.

## 5. Conclusions

In summary, this study demonstrates that plasma-enriched sEVs undergo distinct quantitative and phenotypic changes following SCI, which vary by injury severity and phase. Moderately injured animals exhibited an early increase in circulating sEVs, while severely injured animals showed a blunted response, potentially reflecting differences in cellular viability, spinal vascular disparities, or inflammatory activation. During the chronic phase, vesicle numbers declined overall, yet specific subpopulations, particularly CD41^+^, CD44^+^, and CD61^+^ sEVs, persisted or increased, suggesting ongoing systemic or CNS-specific remodeling processes. These findings support the hypothesis that sEVs act as dynamic reporters and potential modulators of the injury response. Specific surface marker profiles may reflect shifts in immune status, cellular origin, or regenerative signaling, underscoring their potential as non-invasive biomarkers for SCI monitoring. However, several limitations must be acknowledged. First, plasma sEVs represent a heterogeneous population, and this study did not definitively determine the cellular origin of specific sEV subsets. Second, biological variables such as age, which are known to influence both sEV profiles and SCI outcomes, were not consistently controlled in this study [48]; animal age could only be matched across subacute and chronic cohorts at the time of SCI or the time of blood collection, not both. Third, while we minimized contamination by preparing platelet-free plasma, we did not perform red or white blood cell counts in the perfusate blood, factors that could have affected sEV composition and should be considered in future studies, particularly considering common post-injury complications such as hematuria or subclinical infection. Finally, the field continues to face methodological challenges in sEV isolation, quantification, and standardization across platforms. To advance the utility of sEVs as biomarkers or therapeutic tools, future research must prioritize both biological and technical clarity. Understanding the cellular origin and functional roles of sEVs in the context of pathology is essential, as highlighted in this study. Equally important is the adoption of reproducible, standardized, and robust isolation methods as well as comprehensive sample quality controls to ensure consistency across studies and enable clinical translation.

## Data Availability

The raw data supporting the conclusions of this article will be made available by the authors upon request.

## Supplementary Materials

The following supporting information can be downloaded at https://www.mdpi.com/article/10.3390/cells14141065/s1. Figure S1: Flow Cytometry Gating Strategy for MACSPlex Assay Bead Population Analysis; Figure S2: Overall surface marker expression profiles of plasma-derived sEVs across all groups as measured by MACSPlex analysis; Figure S3: Quantification of surface marker expression in plasma-derived sEVs normalized to CD9 (fold change) comparing naïve, subacute, and chronic timepoints following moderate (50 or 60 kdyn) spinal cord injury; Table S1: Description of MACSPlex Surface Markers Included in the Study.
