# Supplementary material for: Temporal and Severity-Dependent Alterations in Plasma Extracellular Vesicle Profiles Following Spinal Cord Injury
Source: Cells. 2025 Jul 11;14(14):1065. doi: 10.3390/cells14141065 (PMC12294027; doi:10.3390/cells14141065)
Supplement: Supplementary file 1 [file cells-14-01065-s001.zip › cells-3705506-supplementary.pdf]

Supplementary data

Supplementary Table S1. Description of MACSPlex Surface Markers Included in the Study

Marker	Description
CD81	A tetraspanin membrane protein commonly enriched on extracellular vesicles (EVs), including exosomes. It plays a role in membrane organization, vesicle formation, and intercellular communication, and is widely used as a canonical EV marker.
CD63	A tetraspanin protein frequently found on the surface of exosomes. It is involved in vesicle trafficking and membrane fusion processes, making it a widely used marker for identifying and isolating extracellular vesicles.
CD9	A tetraspanin protein commonly present on exosomes and other extracellular vesicles, including those derived from platelets. It participates in cell adhesion, signal transduction, and vesicle formation, making it a key marker for EV identification.
CD41	Also known as integrin αIIb, CD41 is a platelet-specific surface glycoprotein involved in platelet aggregation and clot formation. It is commonly used as a marker to identify platelet-derived extracellular vesicles.
CD62P	Also known as P-selectin, CD62P is an adhesion molecule expressed on activated platelets and endothelial cells. It plays a key role in mediating interactions between platelets, leukocytes, and the endothelium, and is used as a marker for platelet activation and platelet-derived extracellular vesicles.
CD29	Also known as integrin β1, CD29 is a cell surface receptor involved in cell adhesion, migration, and signaling. It is broadly expressed on various cell types and is found on extracellular vesicles, where it contributes to vesicle-cell interactions.
CD45	Also known as leukocyte common antigen, CD45 is a transmembrane protein tyrosine phosphatase expressed on all nucleated hematopoietic cells (white blood cells). It is commonly used as a marker to identify leukocyte-derived extracellular vesicles.
CD61	Also known as integrin β3, CD61 is a glycoprotein expressed on platelets and megakaryocytes. It plays a key role in platelet aggregation and blood clotting, and serves as a marker for platelet-derived extracellular vesicles.
CD24	A small glycosylphosphatidylinositol (GPI)-anchored protein expressed on various cell types,

	including hematopoietic and neural cells. It is involved in cell adhesion and signaling, and is used as a marker for certain extracellular vesicle subpopulations.
CD205	Also known as DEC-205, CD205 is a C-type lectin receptor primarily expressed on dendritic cells and some epithelial cells. It is involved in antigen uptake and processing, and can be used as a marker for dendritic cell-derived extracellular vesicles.
CD115	Also known as the macrophage colony-stimulating factor receptor (M-CSFR), CD115 is expressed on monocytes, macrophages, and their precursors. It regulates survival, proliferation, and differentiation of these cells, and serves as a marker for monocyte/macrophage-derived extracellular vesicles.
CD40	A costimulatory protein expressed on antigen-presenting cells such as B cells, dendritic cells, and macrophages. It plays a crucial role in immune activation and inflammation and is used as a marker for extracellular vesicles derived from activated immune cells.
CD86	A costimulatory molecule expressed on antigen-presenting cells, including dendritic cells, macrophages, and B cells. It is involved in T-cell activation and immune response regulation, and serves as a marker for extracellular vesicles from activated immune cells.
CD11b	Also known as integrin αM, CD11b is expressed on myeloid cells such as monocytes, macrophages, neutrophils, and microglia. It plays a role in adhesion, migration, and phagocytosis, and is used as a marker for extracellular vesicles derived from these immune cells.
CD11c	Also known as integrin αX, CD11c is primarily expressed on dendritic cells, some macrophages, and certain subsets of monocytes. It is involved in cell adhesion and antigen presentation and serves as a marker for extracellular vesicles derived from dendritic cells and related immune cells.
CD66a	Also known as CEACAM1 (carcinoembryonic antigen-related cell adhesion molecule 1), CD66a is expressed on epithelial cells and certain immune cells like neutrophils and lymphocytes. It functions in cell adhesion, immune regulation, and signaling, and can be used as a marker for extracellular vesicles from these cell types.
CD2	A cell adhesion molecule expressed primarily on T cells and natural killer (NK) cells. It plays a role in T cell activation and interaction with antigen-presenting cells, and is used as a marker for extracellular vesicles derived from T and NK cells.

CD3	A protein complex expressed on the surface of T cells, essential for T cell receptor (TCR) signaling and T cell activation. It serves as a specific marker for T cell-derived extracellular vesicles.
CD4	A glycoprotein expressed primarily on helper T cells, as well as on monocytes and macrophages. It functions as a co-receptor for MHC class II molecules and is used as a marker for extracellular vesicles derived from these immune cells.
CD8a	A glycoprotein expressed mainly on cytotoxic T cells and some natural killer (NK) cells. It acts as a co-receptor for MHC class I molecules and is used as a marker for extracellular vesicles originating from cytotoxic immune cells.
MHC Class II	Major histocompatibility complex class II molecules are expressed primarily on antigen-presenting cells such as dendritic cells, macrophages, and B cells. They present extracellular antigen peptides to CD4+ T cells and serve as markers for extracellular vesicles derived from these immune cells.
CD69	An early activation marker expressed on activated T cells, B cells, and natural killer (NK) cells. It plays a role in immune response regulation and is used as a marker for extracellular vesicles from activated immune cells.
CD25	Also known as the α-chain of the interleukin-2 receptor (IL-2Rα), CD25 is expressed on activated T cells, regulatory T cells, and some B cells. It is involved in immune regulation and proliferation, serving as a marker for extracellular vesicles from activated and regulatory immune cells.
CD19	A B cell-specific surface protein involved in B cell activation and signaling. It is widely used as a marker for extracellular vesicles derived from B cells.
CD20	A transmembrane protein expressed on mature B cells involved in B cell activation and proliferation. It is commonly used as a marker for extracellular vesicles originating from B cells.
CD31	Also known as platelet endothelial cell adhesion molecule-1 (PECAM-1), CD31 is expressed on endothelial cells, platelets, and certain leukocytes. It plays a role in leukocyte migration and angiogenesis, and serves as a marker for extracellular vesicles derived from these cells.
CD146	Also known as melanoma cell adhesion molecule (MCAM), CD146 is expressed on endothelial cells, some immune cells, and certain tumor cells. It is involved in cell adhesion, migration, and angiogenesis, and serves as a marker for extracellular vesicles from endothelial and related cells.

CD105	Also known as endoglin, CD105 is a component of the TGF-β receptor complex predominantly expressed on endothelial cells. It plays a key role in angiogenesis and vascular development, and is used as a marker for extracellular vesicles derived from endothelial cells.
EphA2	A member of the Eph receptor tyrosine kinase family involved in cell adhesion, migration, and angiogenesis. It is expressed on various cell types, including endothelial and cancer cells, and can be found on extracellular vesicles associated with tumor progression and vascular remodeling.
CD326	Also known as EpCAM (epithelial cell adhesion molecule), CD326 is expressed on epithelial cells and certain carcinoma cells. It functions in cell adhesion and signaling and serves as a marker for extracellular vesicles derived from epithelial tissues and tumors.
Prominin-1	Also known as CD133, Prominin-1 is a pentaspan transmembrane glycoprotein expressed on stem and progenitor cells, including cancer stem cells. It is involved in maintaining stem cell properties and is used as a marker for extracellular vesicles originating from stem-like cells.
CD140a	Also known as platelet-derived growth factor receptor alpha (PDGFRα), CD140a is a receptor tyrosine kinase expressed on mesenchymal cells, including fibroblasts and some progenitor cells. It plays a role in cell growth and development and serves as a marker for extracellular vesicles derived from these cell types.
CD44	A cell surface glycoprotein involved in cell adhesion, migration, and signaling. It is widely expressed on many cell types, including immune cells and cancer cells, and serves as a marker for extracellular vesicles associated with cell communication and tumor progression.
CD49b	Also known as integrin α2, CD49b is expressed on various cell types including natural killer (NK) cells, platelets, and some epithelial cells. It functions in cell adhesion and migration and is used as a marker for extracellular vesicles from these cells.
CD49e	Also known as integrin α5, CD49e is a cell surface receptor involved in cell adhesion and migration by binding to fibronectin. It is expressed on various cell types, including fibroblasts and immune cells, and serves as a marker for extracellular vesicles derived from these cells.
H-2 (MHC I)	The mouse major histocompatibility complex class I molecules, analogous to human MHC I, expressed on almost all nucleated cells. They present endogenous antigen peptides to CD8+ T cells and

	serve as markers for extracellular vesicles from various mouse cell types.
REA Control	A reagent control antibody used as a negative isotype control in flow cytometry to assess non-specific binding and background staining, ensuring specificity of antibody-based detection in extracellular vesicle analysis.
Rat IgG1 Control	An isotype control antibody used in flow cytometry to measure non-specific binding of rat IgG1 antibodies, helping to distinguish specific from background staining in extracellular vesicle or cell surface marker analysis.

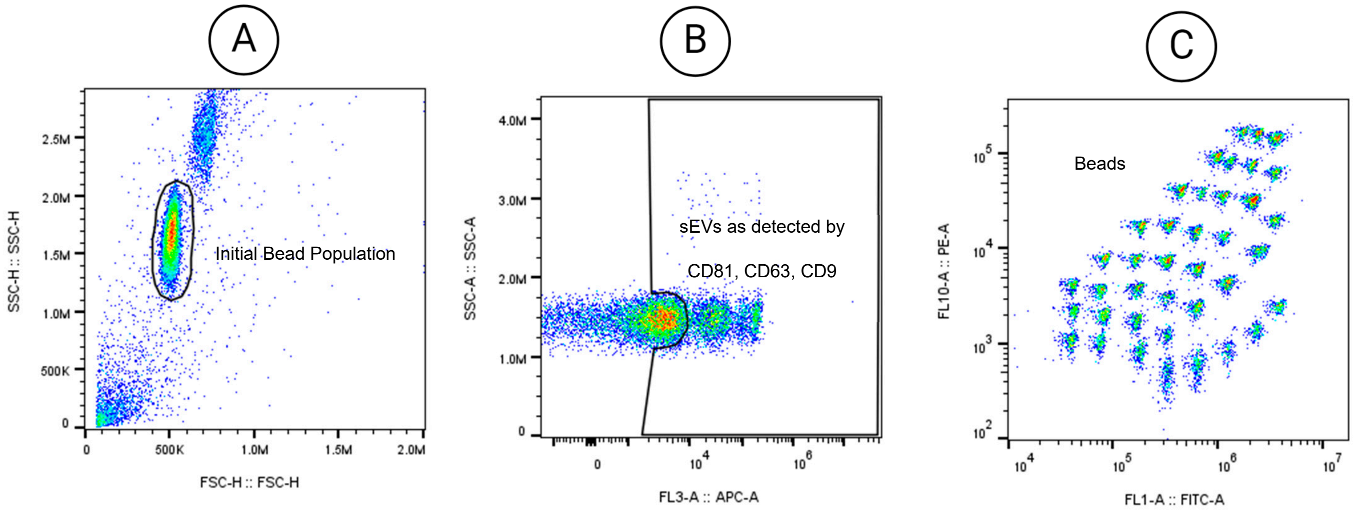

Supplementary Figure S1. Flow Cytometry Gating Strategy for MACSPlex Assay Bead Population Analysis. A *Initial identification of the bead population using side scatter height (SSC-H) versus forward scatter height (FSC-H) gating.* ***B*** *Detection of plasma-derived sEVs bound to tetraspanin markers (CD81, CD63, CD9)* by gating around buffer only control *as shown via SSC-A versus APC-A gating.* ***C*** *individual bead populations based on marker expression using PE-A versus FITC-A gating.*

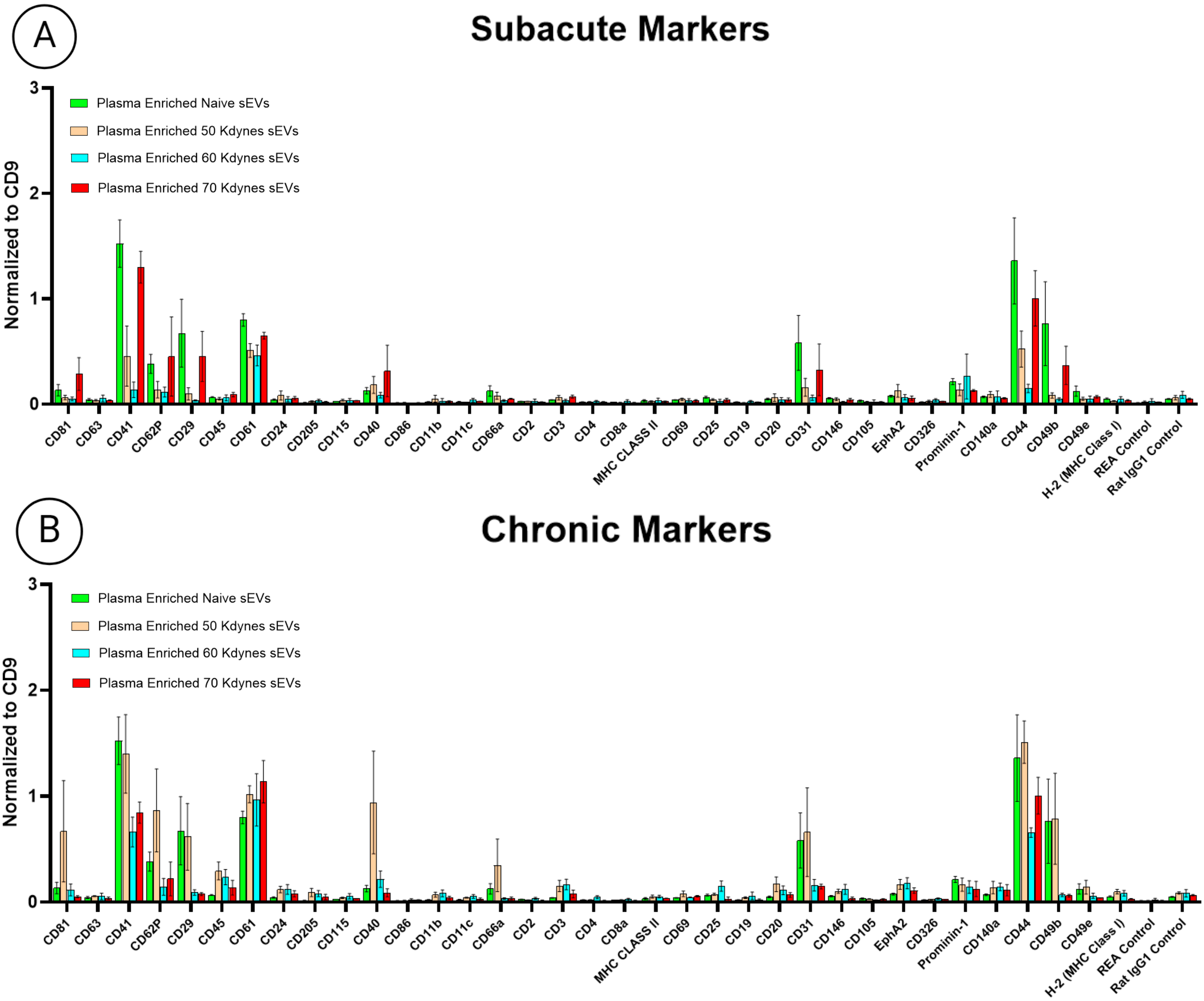

Supplementary Figure S2. Overall surface marker expression profiles of plasma-derived sEVs across all groups as measured by MACSPlex analysis. (A) *Subacute phase.* ***(B)*** *Chronic phase. Data represent median fluorescent intensities normalized to CD9 for each marker. All experiments were performed in triplicate; data are presented as mean ± SEM.*

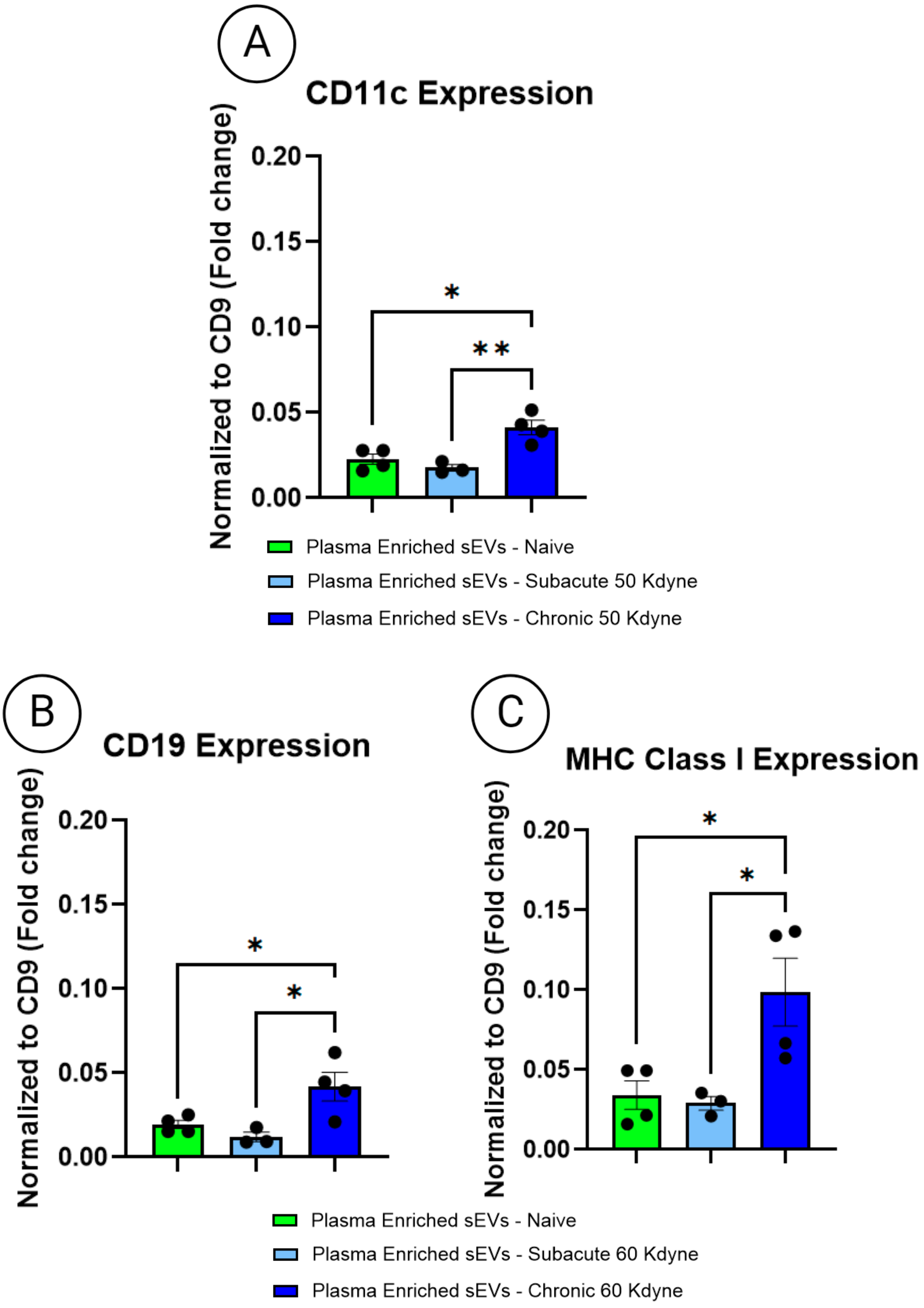

Supplementary Figure S3. Quantification of surface marker expression in plasma-derived sEVs normalized to CD9 (fold change) comparing naïve, subacute, and chronic timepoints following moderate (50 or 60 kdyn) spinal cord injury. **A** *CD11c expression in the 50 kdyn injury group.* **B** *CD19 and MHC Class I expression in the 60 kdyn injury group.* **C** *All data are normalized to CD9 and expressed as fold change. One-way ANOVA followed by Tukey's multiple comparisons test was used to assess statistical significance. All experiments were performed in triplicate; data are presented as mean ± SEM. Significance levels: *p < 0.05, **p < 0.01.*
